# Production of the polyketide 6-deoxyerythronolide B in the heterologous host *Bacillus subtilis*

**DOI:** 10.1007/s00253-015-6990-6

**Published:** 2015-10-02

**Authors:** Jana Kumpfmüller, Karen Methling, Lei Fang, Blaine A. Pfeifer, Michael Lalk, Thomas Schweder

**Affiliations:** Pharmaceutical Biotechnology, Institute of Pharmacy, Ernst-Moritz-Arndt-University, Felix-Hausdorff-Str. 3, 17489 Greifswald, Germany; Institute of Biochemistry, Ernst-Moritz-Arndt-University, Felix-Hausdorff-Str. 4, 17489 Greifswald, Germany; Department of Chemical and Biological Engineering, State University of New York at Buffalo, 904 Furnas Hall, Buffalo, NY 14260-4200 USA; Present Address: Department of Biomolecular Chemistry, Leibniz Institute for Natural Product Research and Infection Biology, HKI, Beutenbergstr. 11a, 07745 Jena, Germany

**Keywords:** *Bacillus subtilis*, Deoxyerythronolide B synthase, Heterologous expression, Polyketide, *acoA* promoter, Metabolic engineering

## Abstract

Polyketides, such as erythromycin, are complex natural products with diverse therapeutic applications. They are synthesized by multi-modular megaenzymes, so-called polyketide synthases (PKSs). The macrolide core of erythromycin, 6-deoxyerythronolide B (6dEB), is produced by the deoxyerythronolide B synthase (DEBS) that consists of three proteins each with a size of 330–370 kDa. We cloned and investigated the expression of the corresponding gene cluster from *Saccharopolyspora erythraea*, which comprises more than 30 kb, in *Bacillus subtilis*. It is shown that the DEBS genes are functionally expressed in *B. subtilis* when the native *eryAI–III* operon was separated into three individual expression cassettes with optimized ribosomal binding sites. A synthesis of 6dEB could be detected by using the acetoin-inducible *acoA* promoter and a fed-batch simulating EnBase-cultivation strategy. *B. subtilis* was capable of the secretion of 6dEB into the medium. In order to improve the 6dEB production, several genomic modifications of this production strain were tested. This included the knockout of the native secondary metabolite clusters of *B. subtilis* for the synthesis of surfactin (26 kb), bacillaene (76 kb), and plipastatin (38 kb). It is revealed that the deletion of the *prpBD* operon, responsible for propionyl-CoA utilization, resulted in a significant increase of the 6dEB product yield when exogenous propionate is provided. Although the presented *B. subtilis* 6dEB production strain is not competitive with established *Escherichia coli* 6dEB production strains, the results of this study indicate that *B. subtilis* is a suitable heterologous host for the secretory production of a complex polyketide.

**Electronic supplementary material:**

The online version of this article (doi:10.1007/s00253-015-6990-6) contains supplementary material, which is available to authorized users.

## Introduction

The broad-spectrum antibiotic erythromycin, produced by the actinomycete *Saccharopolyspora erythraea*, is a well-studied representative of a class of complex natural products called polyketides (Corcoran 1981). Its macrolide core, 6-deoxyerythronolide B (6dEB), is synthesized by a polyketide synthase (PKS) that has emerged as the prototypical modular megasynthase (Khosla et al. 2007).

In general, PKSs can assemble complex biomolecules from simple building blocks such as malonyl coenzym A (malonyl-CoA), methylmalonyl-CoA, and propionyl-CoA through an assembly line thiotemplate mechanism (Cane and Walsh 1999). As a result of the variety of number and type of incorporated units in combination with the possible partial or complete reduction of each keto-function and further (also post-enzymatic) modifications of the metabolite, this group of natural products shows an enormous degree of structural diversity. This in turn results in the extraordinary variety of biological properties and is the reason for the significance of this group, especially for pharmaceutical applications.

Unfortunately, natural sources can in many cases not cover the increasing demand for new bioactive natural products (Koehn and Carter 2005). Due to the structural complexity of polyketides, synthetic routes are precluded and the development of fermentation processes is necessary to allow sufficient production for pre-clinical and clinical studies. Quite often, success is closely linked with the selection of a suitable surrogate host. But, for heterologous polyketide biosynthesis, three challenges have to be addressed. First, the entire gene cluster needs to be functionally transferred. Second, the proteins have to be expressed, correctly folded, and post-translationally modified. To this purpose, a universal phosphopantetheinyl transferase (PPTase) is required for pantetheinylation and thus activation of the PKS (Lambalot et al. 1996). Third, metabolic building blocks must be available. For example, the corresponding gene cluster for 6dEB synthesis comprises three genes of approximately 10 kb (*eryAI–III*). They encode for three large proteins, denoted DEBS1, DEBS2, and DEBS3, that show a size of 330–370 kDa each and form an enzymatic complex. Along this protein complex, there are 28 active sites that are precisely arranged and responsible for the stepwise combination and modification of one propionyl-CoA primer unit and six (2*S*)-methylmalonyl-CoA extender units (Fig. S1 in the Supplementary Material) (Khosla et al. 2007). Furthermore, with regard to the industrial production of drugs, many safety requirements which are regulated and controlled by the European Medicine Agency (EMA) or the Food and Drug Administration (FDA) have to be considered.

The Gram-positive, non-pathogenic strain *Bacillus subtilis* can fulfill these safety requirements as it owns the Generally Recognized As Safe (GRAS) status and the Qualified Presumption of Safety (QPS) certification (Leuschner et al. 2010; Sietske de Boer and Diderichsen 1991). Due to the absence of lipopolysaccharides on the outer cell membrane, which are a typical feature of Gram-negative hosts such as *Escherichia coli* and act as endotoxins, the downstream process is simplified and thus less cost intensive (Petsch and Anspach 2000). Furthermore, because of its natural ability to secrete peptides into the culture environment, which also facilitates downstream processing, *B. subtilis* is one of the most important strains for industrial enzyme production (van Dijl and Hecker 2013).

For heterologous production of secondary metabolites, it is especially noteworthy that *B. subtilis*, in contrast to *E. coli*, is a natural producer of several bioactive compounds (Stein 2005). The antibiotically effective bacillaene, which is synthesized via a PKS/non-ribosomal peptide synthetase (NRPS)-hybrid pathway, is one representative (Patel et al. 1995; Butcher et al. 2007). Two other examples are the non-ribosomal lipopeptide surfactin (Arima et al. 1968; Nakano et al. 1991) and plipastatin (Tsuge et al. 2007) with antimicrobial activities. Furthermore, with the secretory production of the peptide antibiotic bacitracin from *Bacillus licheniformis* (Eppelmann et al. 2001) as well as the cyclohexadepsipeptide enniatin from *Fusarium oxysporum* showing various antimicrobial effects (Zobel et al. 2015), the suitability of *B. subtilis* as a heterologous host for non-ribosomal peptide–type compounds could already be demonstrated.

To evaluate the suitability of *B. subtilis* as a heterologous host for the engineered biosynthesis of polyketides, we chose the already mentioned and well-investigated macrolide 6dEB as a model compound. Since this metabolite needs to be activated by several post-PKS modifications, it does not show any antibiotic effect that could inhibit the host. Furthermore, although heterologous production was already demonstrated in *Streptomyces coelicolor* (Kao et al. 1994) and *E. coli* (Pfeifer et al. 2001, 2002), there was an interest in extending the list of heterologous hosts further in anticipation of future biosynthetic efforts which may benefit from the capabilities of *B. subtilis*.

## Materials and methods

### General information

Unless stated otherwise, all chemicals were purchased from Roth (Karlsruhe, Germany) at the highest purity available and were used without further purification. Oligonucleotides (listed in Table S1 in the Supplementary Material) were synthesized and provided by Life Technologies (Darmstadt, Germany). All plasmids and strains used in this study are listed in Tables 1 and 2, respectively. Relevant translational and transcriptional elements of the native and modified *eryAI–III* gene cassettes are summarized in Table 3. All cloning procedures were carried out in *E. coli* DH10B (Invitrogen, Darmstadt, Germany) [F- *endA1 recA1 galE15 galK16 nupG rpsL* Δl*acX74* Φ80*lacZ*Δ*M15 araD139* Δ(*ara,leu*)7697 *mcrA* Δ(*mrr-hsdRMS-mcrBC*) λ-]. Restriction enzymes and other DNA-modifying enzymes were used as specified by the supplier (New England Biolabs, Frankfurt, Germany). PCR products were purified with the High Pure PCR Product Purification Kit (Roche, Mannheim, Germany). Plasmid isolation was performed using the High Pure Plasmid Isolation Kit (Roche, Mannheim, Germany). For gel extraction, the QIAquick Gel Extraction Kit from Qiagen (Hilden, Germany) was used. Recombinant *B. subtilis* strains were verified by colony PCR as previously described (Kumpfmüller et al. 2013). All plasmid constructs (see Supplementary Material) and chromosomal integrations were verified by sequencing carried out by Eurofins Genomics (Elbersberg, Germany).

**Table 1:** Plasmids used in this study

Plasmid	Function	Reference
P1394	Cosmid carrying wild-type *eryAI–III*-cluster in pWE13	Cosmid library of the Peter Leadlay lab^a^
pAMY-lox-SSS	Integration of genes into the *amyE* locus with *lox*-SSS-cassette	Kumpfmüller et al. (2013)
pAMY-Spec	Integration of genes into the *amyE* locus with remaining Spec^R^-cassette	Kumpfmüller et al. (2013)
pAMY-SSS	Integration of genes into the *amyE* locus with SSS-cassette	Kumpfmüller et al. (2013)
pJET-lox-SSS	Source of *lox*-SSS-cassette	Kumpfmüller et al. (2013)
pJK64a	Reconstitution of genetic *sfp* defect with *lox*-SSS-cassette	Zobel et al. (2015)
pJK93	Deletion of *srfA* operon with remaining Kan^R^-cassette	Zobel et al. (2015)
pJK94	Deletion of *srfA* operon with remaining Spec^R^-cassette	This study
pJK111	Deletion of *srfA* operon with SSS-cassette	This study
pJK119	Integration of *eryAI* with *acoA*-promoter in *srfA* gene locus (Spec^R^-cassette)	This study
pJK119c	Integration of *eryAI* with *acoA*-promoter in *srfA* gene locus (*lox*-SSS-cassette)	This study
pJK123	Deletion of *srfA* operon with remaining Kan^R^-cassette	Kumpfmüller, unpublished
pJK126	Deletion of *srfA* operon with remaining Nm^R^-cassette	Kumpfmüller, unpublished
pJK134	Deletion of *srfA* operon with remaining Kan^R^ and Cm^R^ for RedET cloning	This study
pJK139	Integration of *eryAII* with *acoA*-promoter in *srfA* gene locus (Kan^R^-cassette)	This study
pJK139a	Integration of *eryAII* with *acoA*-promoter in *srfA* gene locus (Kan^R^- and lox-SSS-cassette)	This study
pJK140	Integration of *eryAII* with RBS in *srfA* gene locus (Kan^R^-cassette)	This study
pJK140a	Integration of *eryAII* with RBS in *srfA* gene locus (Kan^R^- and *lox*-SSS-cassette)	This study
pJK155	Integration of wild-type *eryAI–III*-cluster with *acoA*-promoter in *srfA* gene locus	This study
pJK179	Deletion of *pksX* operon	Zobel et al. (2015)
pJK191	Deletion of *srfA* operon	Zobel et al. (2015)
pJK205	Insertion in *lytC* gene locus	Zobel et al. (2015)
pJK206	Deletion of *srfA* operon with Kan^R^- and *lox*-SSS-cassette	This study
pJK209	Deletion of *spoIIGA* gene	Zobel et al. (2015)
pJK219	Toolbox-plasmid for integration of CDS with *acoA*-promoter in *srfA* gene locus (*lox*-SSS-cassette)	This study
pJK226	Deletion of restriction and modification system	Zobel et al. (2015)
pJK245	Integration of *eryAIII* with *acoA*-promoter in *srfA* gene locus	This study
pJK246	Integration of *eryAIII* with RBS in *srfA* gene locus	This study
pJK254	Deletion of *pps* operon	This study
pJK257	Integration of *ery-orf5* with *acoA*-promoter in *srfA* gene locus	This study
pJK258	Integration of *ery-orf5* with *acoA*-promoter in *srfA* gene locus, in addition to *eryAI–III*-cluster	This study
pJK260	Deletion of *prp* operon	This study
pMSE3	High-copy *E. coli*/*B. subtilis* shuttle vector with Kan^R^-cassette	Silbersack et al. (2006)

*Cm* ^*R*^ chloramphenicol resistance cassette, *Kan* ^*R*^ kanamycin resistance cassette, *Nm* ^*R*^ neomycin resistance cassette, *Spec* ^*R*^ spectinomycin resistance cassette, *ss* six site, *SSS* Spec^R^ flanked by two *ss*, *lox*-*SSS* SSS surrounded by a *lox71* and *lox66* site

^a^The cosmid P1394 was received from the cosmid library of the Peter Leadlay laboratory from the Department of Biochemistry at the University of Cambridge. The cosmid sequence with the *eryAI–III*-cluster can be found on the “*Saccharopolyspora erythraea* genome project web site” at http://131.111.43.95/gnmweb/index.html.

**Table 2:** Strains used in this study

Strain	Relevant genotype	Reference
*B. subtilis* 168	Wild type, sfp^0^	Zeigler et al. (2008)
*B. subtilis* JK13	*ΔsacA::(Zeo* ^*R*^ *, P*_*spac*_ *-comS, lacI, P*_*xylA*_ *-cre, xylR)*	Zobel et al. (2015)
*B. subtilis* JK34	*BsJK13 + ΔsrfA::comS, Kan* ^*R*^ *,lox72*	This study
*B. subtilis* JK47	*BsJK34 + RM::lox72*	This study
*B. subtilis* JK53	*BsJK34 + ΔsrfA:: (P*_*acoA*_ *-eryAI-T7, lox72)*	This study
*B. subtilis* JK54	*BsJK53 + RM::lox72*	This study
*B. subtilis* JK57	*BsJK47 + ΔsrfA::(P*_*acoA*_ *-eryAI-T7, P*_*acoA*_ *- eryAII, Kan* ^*R*^ *, lox72)*	This study
*B. subtilis* JK58	*BsJK47 + ΔsrfA: (P*_*acoA*_ *-eryAI-T7, P*_*acoA*_ *- eryAII -T7, P*_*acoA*_ *- eryAIII-T7, lox72)*	This study
*B. subtilis* JK59	*BsJK58 + sfp* ^*+*^ *::lox72*	This study
*B. subtilis* JK59-1	*BsJK59 + ΔamyE::lox72*	This study
*B. subtilis* JK60	*BsJK59-1 + ΔsrfA::( P*_*acoA*_ *-eryAI-T7, P*_*acoA*_ *- eryAII -T7, P*_*acoA*_ *- eryAIII-T7, lox72, P*_*acoA*_ *-ery-orf5-T7)*	This study
*B. subtilis* JK62	*BsJK60 + ΔlytC::lox72*	This study
*B. subtilis* JK63	*BsJK62 + ΔspoIIGA::lox72*	This study
*B. subtilis* JK64	*BsJK47 + ΔsrfA:: (P*_*acoA*_ *-eryAI-RBS-eryAII, Kan* ^*R*^ *, lox72)*	This study
*B. subtilis* JK65	*BsJK47 + ΔsrfA:: (P*_*acoA*_ *-eryAI-RBS-eryAII-RBS-D3-T7, lox72)*	This study
*B. subtilis* JK66	*BsJK65 + sfp* ^*+*^ *::lox72*	This study
*B. subtilis* JK66-1	*BsJK66 + ΔamyE::lox72*	This study
*B. subtilis* JK68	*BsJK63 + ΔpksX::lox72*	This study
*B. subtilis* JK70	*BsJK47 + ΔsrfA:: (P*_*acoA*_ *-eryAI–III*_*nat*_ *-T7, ss)*	This study
*B. subtilis* JK71	*BsJK70 + sfp* ^*+*^ *::lox72*	This study
*B. subtilis* JK71-1	*BsJK71 + ΔamyE::lox72*	This study
*B. subtilis* JK84	*BsJK13 + RM::lox72, sfp* ^*+*^ *::lox72, ΔsrfA::comS-lox72, ΔpksX::lox72, ΔspoIIGA::lox72, ΔlytC::lox72, Δpps::lox72, ΔamyE::lox72*	Kumpfmüller, unpublished
*B. subtilis* JK120	*BsJK68 + Δpps::lox72*	This study
*B. subtilis* JK125	*BsJK120 + Δprp::lox72*	This study

*Kan* ^*R*^ kanamycin resistance cassette, *Spec* ^*R*^ spectinomycin resistance cassette, *Zeo* ^*R*^ zeocin resistance cassette, *ss* six site, *lox72 lox72* site

**Table 3:** Transcriptional and translational elements of the native and the modified *eryAI–III* gene cassettes

Strain	mRNA	Gene	RBS	← Distance →	Start codon	Stop codon
*B. subtilis* JK59-1	monocistronic	*eryAI*	GGAGGA	7 bp	ATG	TAA
		*eryAII*	GGAGGA	7 bp	ATG	TAA
		*eryAIII*	GGAGGA	7 bp	ATG	TAA
*B. subtilis* JK66-1	tricistronic	*eryAI*	GGAGGA	7 bp	ATG	TAA
		*eryAII*	GGAGGA	7 bp	ATG	TAA
		*eryAIII*	GGAGGA	7 bp	ATG	TAA
*B. subtilis* JK71-1	tricistronic	*eryAI*	GGAGGA	7 bp	ATG	TGA
		*eryAII*	TGGAGA	4 bp	GTG	TAG
		*eryAIII*	AGAGGA	7 bp	ATG	TAA

### Construction of strains

In this study, an optimized protocol for rapid and multiple genome modification of *B. subtilis* was used which was previously described (Kumpfmüller et al. 2013). For *comS* induction, 100 μM isopropyl-β-D-thiogalactoside (IPTG) was added when the cells were diluted. Unless stated otherwise, the antibiotic selection marker was removed after successful chromosomal integration using a method based on the recombination of flanking *lox* sites with chromosomally localized *cre* as described in the above mentioned protocol. For *B. subtilis*, antibiotics were used in the following final concentrations: 100 μg/mL spectinomycin, 20 μg/mL kanamycin, and 20 μg/mL zeocin.

### Media and cultivation

For expression studies, *B. subtilis* strains were cultivated in the fed-batch simulating EnBase® system from BioSilta. To this purpose, the *EnPresso B Tablet Cultivation Set* was used in combination with 24-well deepwell culture plates and airporous seals for multi-well plates (all BioSilta, Oulu, Finland). For cultivation, 2 mL of prepared medium (including 0.1 % acetoin for promoter induction, 1.5 U/L Reagent A for enzyme-based substrate delivery, 20 μg/mL zeocin for selection, and 20 mM sodium propionate as 6dEB precursor, if not indicated otherwise) was inoculated with 100 μL of a late-exponential pre-culture (LB medium; 37 °C, OD_600_ approx. 2.0) and incubated at 30 °C at 250 rpm (25-mm amplitude). After overnight culture (16 h), 200 μL of the dissolved booster solution and another 1.5 U/L Reagent A were added and the cultivation was continued for 48 h under shaking conditions.

### RNA isolation and analysis

RNA isolation was done as previously described (Welsch et al. 2012). For slot blot analyses, the Bio-Dot SF Microfiltration Apparatus (Bio-Rad, Munich, Germany) was used according to the manufacturer’s protocol. RNA probes were prepared using the DIG RNA Labeling Kit (SP6/T7) from Roche Life Science (Mannheim, Germany) and the following primers: *eryAI*, 5056/5490; *eryAII*, 5105/5496; and *eryAIII*, 5492/5493. Hybridization and detection were performed as described elsewhere (Welsch et al. 2012).

### Extraction

After cultivation, the cells were harvested and 1300 μL of the supernatant was supplemented with 13 μL of internal standard mixture (1 ng/μL sulfadimethoxine, 2 ng/μL sulfachloropyridazine in methanol, HPLC grade). Subsequently, samples were extracted with an equal volume of ethyl acetate and agitated for 10 min at 30 °C. Phase separation was performed via centrifugation at room temperature and 8500 rpm for 5 min. After ethyl acetate was evaporated under vacuum, the extracts were dissolved in 75 μL HPLC-grade methanol for HPLC-mass spectrometry (MS) analysis.

### HPLC and mass spectrometry

All experiments were carried out on an Agilent 1200 HPLC system coupled to a 6460 Triple Quadrupole mass spectrometer equipped with a Jet stream ESI-source (Agilent Technologies, Waldbronn, Germany). The chromatographic separation was achieved temperature controlled at 25 °C on a Synergi Fusion-RP column (2.5 μm, 50 × 2.0 mm) equipped with a pre-column of the same material (4 × 2.0 mm) both from Phenomenex (Aschaffenburg, Germany). A gradient of mobile phase A (0.1 % formic acid) and mobile phase B (acetonitrile) was used as shown in Table S2 in the Supplementary Material (flow rate of 0.5 mL/min, injection volume of 10 μL). The MS was operated in positive ion mode with multi-reaction monitoring (MRM). The MS/MS fragmentation pattern of 6dEB and the internal standard compounds were determined and the MS parameters were optimized. The optimized source parameters are displayed in Table S3 in the Supplementary Material.

6dEB was monitored with transitions *m*/*z* 409.1 to *m*/*z* 311.2 for relative quantification and *m*/*z* 409.1 to *m*/*z* 391.2 and *m*/*z* 293.2 for identification. Collision energies for the transitions of 6dEB were 25, 21, and 25 V, respectively. Data was acquired and evaluated using the Mass Hunter software; quantitative analysis was done using Mass Hunter Quantitative Analysis (version B03.02, Agilent Technologies, Waldbronn, Germany).

### 6dEB quantification

For quantification of 6dEB titers, the culture volume was increased to 100 mL, and 500-mL Ultra Yield Flasks with the according AirOTop Seals (BioSilta, Oulu, Finland) were used. Supplementation, induction, inoculation, and cultivation were performed as mentioned above. Metabolites form these cultures were extracted twice with 100 mL ethyl acetate and dried with sodium sulfate afterward. After evaporation of the solvent, the extracts were quantified as described elsewhere (Zhang et al. 2015).

## Results

### Construction of the DEBS expression strains

All plasmids for integration of the *eryAI–III* genes are presented in Fig. S2 in the Supplementary Material. The construction of these plasmids is explained in the Supplementary Material text and by Fig. S3 in the Supplementary Material. The starting strain used in this study was *B. subtilis* JK13 (Zobel et al. 2015), a derivative of the Marburg 168 strain, which already contained an IPTG-inducible second gene copy of the competence factor ComS for enhanced transformation efficiency and the xylose-inducible *cre* gene for marker removal.

In order to investigate the expression of the *eryI-III* gene cluster from *Saccharopolyspora erythraea*, which comprises more than 30 kb, in *B. subtilis*, the DEBS genes were localized to the *B. subtilis* JK13 chromosome in three different cluster organizations: (A) native operon, (B) native operon with optimized ribosomal binding sites, and (C) the three native *ery* gene sequences as separate cassettes with optimized ribosomal binding sites. The operons and the individual gene cassettes were set under control of the acetoin inducible *acoA* promoter.

**Fig. 1:**
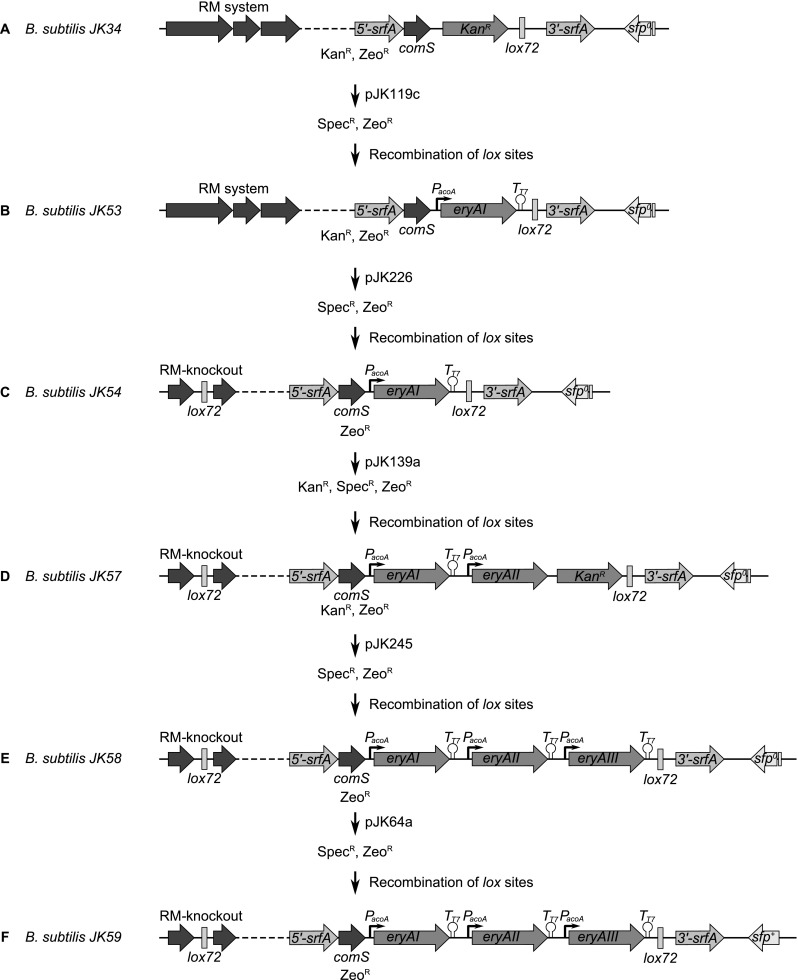
Integration of *eryAI*, *eryAII*, and *eryAIII* as three individually controlled genes with optimized RBS in *B. subtilis* JK34*.* Schematic diagram (not to scale) showing the construction of *B. subtilis* JK59. **a** The former *srfA* gene locus in the chromosome of *B. subtilis* JK34. Transformation of this strain with pJK119c and subsequent recombination of the *lox* sites via Cre resulted in *B. subtilis* JK53 (**b**). Deletion of the RM system via pJK226 resulted in *B. subtilis* JK54 (**c**). Stepwise chromosomal integration of the *eryAII* and *eryAIII* genes under control of the *acoA* promoter via pJK139a and pJK245 resulted in *B. subtilis* JK57 (**d**) and *B. subtilis* JK58 (**e**), respectively. In a final step, the frame shift mutated *sfp* ^*0*^ gene was chromosomally replaced by the native *sfp* ^*+*^ gene by using pJK64a to give *B. subtilis* JK59 (**f**). Thus, PPTase activity of Sfp was reconstituted

Due to a frame shift mutation in the PPTase *sfp* gene, *B. subtilis* 168 strains are not able to activate NRPS and PKS enzymes (Mootz et al. 2001). Hence, they do not produce hemolytic surfactin and deletion of the corresponding *srfA* operon cannot be detected on sheep blood agar plates. In order to allow simple screening for positive transformants after chromosomal integration of the *eryAI–III* genes in the *srfA* gene locus, first a Kan^R^-cassette was inserted by chromosomal integration of pJK206 in *B. subtilis* JK13. The resulting strain *B. subtilis* JK34 was then treated with linearized pJK119c to chromosomally integrate the *P*_*acoA*_-*eryAI*-T_T7_-operon thereby replacing the Kan^R^ gene. Thus, the obtained *eryI*-positive colonies could be screened for loss of kanamycin resistance. For further integration of the *eryAII* gene, the restriction and modification (RM) system (namely, *ydzT–W*, *ydiP–S*, and *ydjA–C*) of the resulting strain (*B. subtilis* JK53) had to be deleted by chromosomal integration of pJK226 to give *B. subtilis* JK54. The positive effect of this knockout (higher transformation efficiency with large DNA fragments) was already described elsewhere (Choi et al. 2009; Haima et al. 1987). Hereafter, *B. subtilis* JK54 was transformed with plasmid pJK139a, resulting in *B. subtilis* JK57. Thereby, a Kan^R^-cassette was introduced together with the *P*_*acoA*_-*eryAII*-operon allowing screening for another resistance switch after the following chromosomal integration of the *P*_*acoA*_-*eryAIII*-T_T7_-operon by using pJK245. Hence, the resulting strain *B. subtilis* JK58 harbored all three *eryA* genes as distinct cassettes, each under the control of the *acoA* promoter (Ali et al. 2001). In a final step, *sfp* activity was reconstituted by chromosomal integration of pJK64a, giving *B. subtilis* JK59. The stepwise construction of this strain is shown in Fig. 1.

For chromosomal integration of the modified *eryAI–III* operon with optimized ribosomal binding sites, *B. subtilis* JK54 was used (see also Fig. 1). The RBS-*eryAII*-cassette was chromosomally introduced together with a Kan^R^-cassette by using pJK140a and thereby removing the T7-terminator of the *P*_*acoA*_-*eryAI*-T_T7_-operon resulting in *B. subtilis* JK64. Hereafter, pJK246 was used for chromosomal integration of the *eryAIII* gene with optimized RBS to complete the *P*_*acoA*_-*eryAI*-RBS-*eryAII*-RBS-*eryAIII*-T_T7_-operon. Due to the subsequent deletion of the Kan^R^-cassette the obtained colonies could be screened for antibiotic resistance switch. For reconstitution of the required *sfp* gene, this strain (*B. subtilis* JK65) was then transformed with pJK64a to give *B. subtilis* JK66 (Fig. S4 in the Supplementary Material).

A chromosomal integration of the 30-kb large wild-type *eryAI–III*-cluster could be done in a single step only after deletion of the RM system. Thus, pJK226 was used to transform *B. subtilis* JK34 to give the kanamycin-resistant *B. subtilis* JK47 strain. Hereafter, this mutant strain was treated with pJK155 thereby replacing the Kan^R^-cassette. Positive clones could be screened for the selection marker switch. For subsequent marker removal, recombination of the flanking six-sites with a plasmid-coded β-recombinase was performed as described elsewhere (Kabisch et al. 2012) resulting in *B. subtilis* JK70, which carries the *P*_*acoA*_-*eryAI–III*_*nat*_-T_T7_-operon. Again, this strain was then transformed with pJK64a for *sfp* reconstitution giving *B. subtilis* JK71 (Fig. S5 in the Supplementary Material).

Since the fed-batch simulating EnBase® system, which was used for expression studies, is based on the enzymatic release of glucose from a polymer, the *amyE* gene coding for the α-amylase had to be knocked out to avoid interference with the tightly controlled substrate delivery by an artificially added amylase. For this purpose, pAMY-lox-SSS was used to chromosomally delete the *amyE* locus of *B. subtilis* JK59, JK66, and JK71 to give the amylase-negative strains *B. subtilis* JK59-1, JK66-1, and JK71-1, respectively.

### Cultivation of DEBS expression strains and detection of 6dEB production

**Fig. 2:**
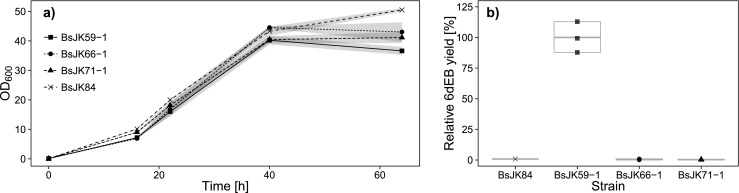
Comparison of engineered *B. subtilis* strains carrying the DEBS genes in different cluster organizations. **a** Growth curves (OD_600_) during cultivation for 64 h (16 h pre- plus 48 h post-boostering) in EnBase® medium. **b** Quantitative MRM analysis of secreted 6dEB of modified *B. subtilis* strains (*t* = 48 h after boostering). BsJK59-1: three individually controlled genes; BsJK66-1: one operon with modified RBS; BsJK71-1: wild-type operon; BsJK84: negative control. *N* = 3, therefore no quartiles, but the median is shown

All three expression strains were cultivated under fed-batch simulating conditions and showed a similar growth behavior that differed from the negative control beginning from *t* = 40 h (24 h post-boostering, see Fig. 2a). This observed reduced cell growth could be a hint for the induced gene expression in these recombinant strains. To qualify specific messenger RNA (mRNA) production, RNA isolation followed by a slot blot analysis was performed. The results indicated that the *eryAI–III* genes were transcribed in all three *B. subtilis* strains independent of the cluster organization (Fig. S6 in the Supplementary Material). However, significant 6dEB production could only be detected for BsJK59-1 which harbored the DEBS genes in three separate cassettes (Fig. 2b). For this strain, it could also be revealed that relative 6dEB yield was more than doubled by prolongation of the EnBase-based cultivation to 48 h after boostering (data not shown). Furthermore, it could be shown that the metabolite is completely secreted, because no 6dEB could be detected in an extract obtained from the cell pellet treated by sonification (data not shown).

### Optimization of 6dEB production in *B. subtilis* BsJK59-1 by engineering the genetic background

For potential optimization of 6dEB production, further genome modifications were investigated. First, the DEBS-specific type II thioesterase (TEII) encoded by the *ery-orf5* gene was chromosomally integrated in *B. subtilis* JK59-1 by using pJK258 to give *B. subtilis* JK60. This accessory enzyme is known to increase 6dEB production in the native producer as well as in the surrogate *E. coli* host (Pfeifer et al. 2002). For enhanced cell growth during heterologous protein production, this strain was then transformed with pJK205 to generate a *lytC* knockout mutant *B. subtilis* JK62 (Kabisch et al. 2012). Hereafter, *spoIIGA* (involved in sporulation) was deleted by chromosomal integration of pJK209 to give the sporulation-deficient strain *B. subtilis* JK63. In order to reduce the metabolic burden of the host, two gene clusters involved in secondary metabolite production were removed from the chromosome in addition to the *srfAA–D* operon. To this purpose, the *pksA–R* operon (~76 kb) was deleted by using pJK179 resulting in *B. subtilis* JK68, which was not able to produce the polyketide bacillaene. In a next step, this strain was transformed with pJK254 to give *B. subtilis* JK120 with a deletion of the 38 kb *ppsA–E* cluster, coding for the plipastatin synthetase (NRPS).

**Fig. 3:**
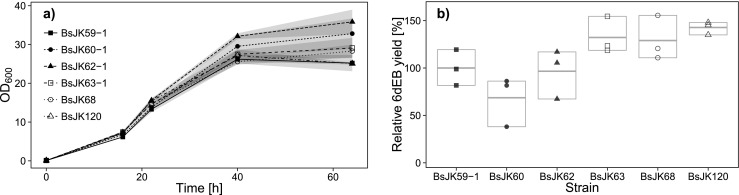
Comparison of modified *B. subtilis* JK59-1 strains with engineered genetic background. **a** Growth curves (OD_600_) during cultivation for 64 h (16 h pre- plus 48 h post-boostering) in EnBase® medium. **b** Quantitative MRM analysis of secreted 6dEB of modified *B. subtilis* strains (*t* = 48 h after boostering). BsJK59-1: three individually controlled genes with optimized RBSs; BsJK60: additional expression of *ery_orf5* (TE II); BsJK62: with *lytC* inactivation; BsJK63: with *spoIIGA* inactivation; BsJK68: deletion of bacillaene synthase cluster; BsJK120: deletion of plipastatin synthase cluster. *N* = 3, therefore no quartiles, but the median is shown

The highest growth rate could be detected for *B. subtilis* JK62 (DEBS1–3, TEII, Δ*lytC*), followed by *B. subtilis* JK60 (DEBS1–3, TEII). *B. subtilis* JK63 (DEBS1–3, TEII, Δ*lytC*, Δ*spoIIGA*) and JK68 (DEBS1–3, TEII, Δ*lytC*, Δ*spoIIGA*, Δ*pksX*) both resulted in a similar cell density compared to the control strain JK62. It is interesting to note that the unmodified 6dEB producer *B. subtilis* JK59-1 and the *B. subtilis* JK120 strain, showing all mentioned modifications, revealed the lowest maximal optical densities (see Fig. 3a).

According to the productivity, the relative 6dEB yield was reduced from 100 % in *B. subtilis* JK59-1 to 69 % in *B. subtilis* JK60 by introduction of the TEII. The additional deletion of *lytC* resulted in the compensation of this negative effect, whereby *B. subtilis* JK62 reached a similar relative 6dEB yield. The *spoIIGA* knockout in *B. subtilis* JK63 resulted in a further enhanced productivity (132 %), which was not affected by the additional deletion of the *pksA–R* operon in *B. subtilis* JK68 (129 %). Best results could be obtained with *B. subtilis* JK120 that also showed the deletion of the *ppsA–E* operon and yielded 143 % (Fig. 3b).

### Influence of the modification of the propionyl-CoA metabolism

To optimize the propionyl-CoA metabolism with regard to the 6dEB production, the *prpBD* operon in *B. subtilis* JK120 responsible for propionyl-CoA utilization was deleted by using pJK260, resulting in *B. subtilis* JK125. In addition to this modification, the removal of propionate, which had always been provided as supplementation, was also investigated.

**Fig. 4:**
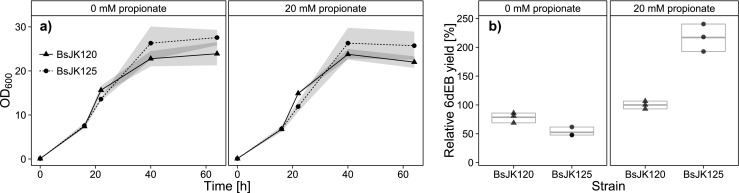
Influence of the *prpBD* knockout and feeding of sodium propionate. **a** Growth curves (OD_600_) during cultivation for 64 h (16 h pre- plus 48 h post-boostering) in EnBase® medium. **b** Quantitative MRM analysis of secreted 6dEB of modified *B. subtilis* strains (*t* = 48 h after boostering). BsJK120: *prpBD* positive control; BsJK125: deletion of *prpBD* operon. *N* = 3, therefore no quartiles, but the median is shown

In general, the cell growth was slightly increased during the cultivation without propionate. Furthermore, the *B. subtilis* JK125 strain reached higher cell densities compared to *B. subtilis* JK120 (Fig. 4a).

Supplementing the medium with 20 mM sodium propionate resulted in an increase in the relative 6dEB yield of 20 % for *B. subtilis* JK120 and 75 % in case of the *prpBD* mutant strain *B. subtilis* JK125. In addition, in the presence of propionate, the relative 6dEB yield in *B. subtilis* JK125 was increased 2.5-fold compared to that in *B. subtilis* JK120. In contrast, the deletion of the *prpBD* operon resulted in a reduced 6dEB productivity (−30 %) in the unsupplemented medium (Fig. 4b).

*B. subtilis* JK125, which has emerged as the best producer, was chosen to quantify the level of 6dEB production. Without further optimization of the culture conditions, a final 6dEB concentration of 2.6 ± 0.3 μg/L was reached with this strain.

## Discussion

Although the DEBS genes could be localized in the chromosome of *B. subtilis* in three different cluster organizations (A, native operon; B, modified native operon with optimized RBS; and C, three separately transcribed genes with optimized RBS) and specific mRNAs of *eryAI*, *eryAII*, and *eryAIII* could be identified in all three expression strains, heterologous 6dEB production could only be detected in the strain *B. subtilis* JK59-1, which controls the expression of the *eryI-III* genes by three separate *acoA* promoters and optimized ribosomal binding sites. The *ery* mRNA analyses indicate that not the transcription but the translation of the mRNAs is the critical step in the heterologous expression of the 6dEB gene clusters. It can be assumed that the monocistronic organization of the *eryAI*, *eryAII*, and *eryAIII* genes in JK59-1 is the main reason for the positive 6dEB synthesis as this is the only distinction to *B. subtilis* JK66-1, which exhibits a tricistronic mRNA and no detectable 6dEB titers. It could be speculated that a greater instability or unfavorable secondary structures of the 30-kb tricistronic mRNA in contrast to the 10-kb monocistronic mRNAs result in insufficient translational initiation and/or unbalanced expression levels of DEBS1, DEBS2, and DEBS3.

It is also demonstrated that only a fed-batch cultivation strategy with the chosen *acoA* promoter system resulted in a significant 6dEB biosynthesis. On the one hand, this might be an effect of the higher cell densities that could be reached in the fed-batch simulating EnBase medium. However, it is more likely that it is a result of the linear cell growth that does not cause oxygen limitation, overflow metabolism, and pH drop (Panula-Perälä et al. 2008). This, in turn, leads to higher protein expression in combination with enhanced metabolic activity (Ukkonen et al. 2011). Thus, a higher synthesis rate of the metabolite can be achieved. This could, for example, already be demonstrated by the increased heterologous production of valinomycin in *E. coli* (Li et al. 2014).

Furthermore, when using *B. subtilis* JK59-1, it could be shown that the 6dEB metabolite accumulates during the last 24 h of cultivation. The productivity of the heterologous host even rose since 6dEB titers could be more than doubled during that time with consistent cell density. This indicates that the metabolite synthesis is not limited by the depletion of one of the substrates and that the DEBS proteins are expressed at a constant level.

In addition, 6dEB was exclusively detectable in the medium but not in the cell extract. Thus, the metabolite seems to be completely secreted into the supernatant. It is noteworthy that such a secretory production is advantageous, especially for simplified detection and downstream processing.

To enhance 6dEB synthesis, the DEBS-specific TEII encoded by *ery-orf5* was chromosomally integrated in *B. subtilis* JK59-1. In general, the function of these discrete hydrolytic enzymes, which are quite often associated with PKS and NRPS gene clusters, is to remove aberrant residues blocking the megasynthase, to participate in substrate selection as well as to release intermediates and products (Kotowska and Pawlik 2014). In recent studies, it could be shown that the deletion of the DEBS-TEII gene in the native host *Saccharopolyspora erythraea* led to both an increase of the side product 8,8′-deoxyoleandolide (15-nor-6-deoxyerythronolide B) and lower erythromycin production (Hu et al. 2003). Furthermore, coexpression of this TEII in a 6dEB producing *E. coli* strain resulted in a doubled product yield (Pfeifer et al. 2002). However, in contrast to our expectations, *B. subtilis* JK60, which harbored the *ery-orf5* gene under the control of the *acoA*-promoter, showed a reduced 6dEB synthesis (−30 %) compared with the parental strain *B. subtilis* JK59-1. The background of this negative effect is not clear, and further investigations are necessary to gain better insight into this unexpected outcome.

Corresponding to the optimization of *B. subtilis* ATCC 6051 as an expression host (Kabisch et al. 2012), *lytC* (the major autolysin) as well as *spoIIGA* (involved in sporulation) was deleted to improve the growth behavior. As expected, the *lytC* knockout mutant *B. subtilis* JK62 showed a higher cell density compared to the parental strain *B. subtilis* JK60 and resulted in an enhanced 6dEB production (+30 %). Production levels could be further increased by the additional deletion of *spoIIGA*, although this knockout led to a reduced cell growth. The enhancement of the 6dEB synthesis (+30 %) in *B. subtilis* JK63 could be an unspecific effect of optimized cell metabolic processes due to the prevention of spore formation (Kabisch et al. 2012).

In addition to these growth-related modifications, the deletion of host-own PKS and NRPS gene clusters was performed in order to reduce the metabolic burden and potential cross talk. The *srfAA–D* genes (~26 kb) responsible for surfactin production (NRP) were already removed by the chromosomal integration of the DEBS operon. Thus, the influence of this knockout on 6dEB production cannot be assessed. Contrary to our expectations, the deletion of the bacillaene synthase cluster (*pksA–R*, ~76 kb) in *B. subtilis* JK68 did not enhance the product yield compared to its parental strain JK63. This might be due to different substrates, which are necessary for 6dEB (propionyl- and methylmalonyl-CoA) and bacillaene (mainly malonyl-CoA) synthesis, whereby no direct competition for starter and extender units exists. The *ppsA–E* knockout mutant *B. subtilis* JK120 also showed no significant increase in 6dEB formation compared to the JK68 control strain. Although the removal of genes of the secondary metabolism did not enhance cell growth and has only a weak effect on 6dEB synthesis, these deletions might be of particular interest for an industrial production strain. Due to safety reasons and simplification of the downstream process, “clean” hosts which do not produce unwanted side products are preferred.

**Fig. 5:**
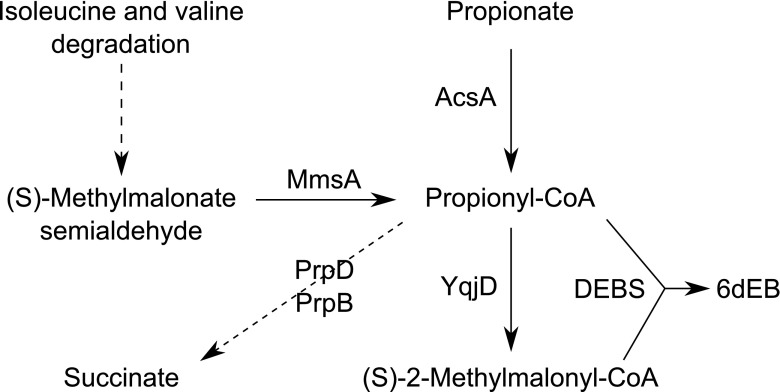
Metabolic pathways of *B. subtilis:* connecting the propionate metabolism to heterologous 6dEB production. The map has been constructed from the KEGG Pathway Database [http://www.genome.jp/kegg/pathway.html#metabolism]

As already mentioned, propionyl- and (2S)-methylmalonyl-CoA are necessary for 6dEB synthesis. *B. subtilis* is capable of propionyl-CoA synthesis either from the isoleucine and valine degradation pathway or directly from propionate (Fig. 5). The starter unit propionyl-CoA can be either metabolized to succinate (via PrpB and PrpD) or converted to (2S)-methylmalonyl-CoA. Our data indicate that *B. subtilis* provides a significant propionyl- and methylmalonyl-CoA pool, but the native precursor concentration is not sufficient for an optimal 6dEB production. Thus, propionate feeding was not mandatory but conductive, and the positive effect of this supplementation could be enhanced by the deletion of the propionyl-CoA degradation pathway. Both procedures resulted in a better propionyl-CoA availability which in turn led to higher intracellular (2S)-methylmalonyl-CoA levels and thus to a more efficient 6dEB biosynthesis.

On the one hand, DEBS is one of the best investigated PKS systems (Khosla et al. 2007). Since the substrates, the structure of the enzymatic complex, and the mechanism of the biosynthesis of 6dEB are known, it is a suitable candidate for a reference system. On the other hand, due to its complexity (28 active sites distributed among three linked proteins) and the size of the corresponding gene cluster (~30 kb), cloning and heterologous expression are challenging. Nevertheless, it is demonstrated that *B. subtilis* is able to functionally express the *eryAI–III* genes, when the three DEBS genes with optimized RBS were individually expressed by separate promoters under fed-batch cultivation conditions. Although the final 6dEB production titer is not comparable with those reached by optimized *E. coli* strains (Zhang et al. 2010), this study indicates that *B. subtilis* is a suitable host for the secretory production of a complex polyketide. To our knowledge, this is the first communication for the heterologous production of a polyketide in the Gram-positive bacterium *B. subtilis*.

## Electronic supplementary material

ESM 1 (PDF 1424 kb)
