# Supplementary material for: Production of the polyketide 6-deoxyerythronolide B in the heterologous host Bacillus subtilis
Source: Appl Microbiol Biotechnol. 2015 Oct 2;100:1209–20. doi: 10.1007/s00253-015-6990-6 (PMC4717160; doi:10.1007/s00253-015-6990-6)
Supplement: Supplementary file 1 — (PDF 1424 kb) [file 253_2015_6990_MOESM1_ESM.pdf]

Applied Microbiology and Biotechnology

Supplementary Material for

Production of the polyketide 6-deoxy-erythronolide B in the heterologous host *Bacillus subtilis*

Jana Kumpfmüller[a$], Karen Methling[b], Lei Fang[c], Blaine A. Pfeifer[c], Michael Lalk[b] and Thomas Schweder[a*]

[a]Pharmaceutical Biotechnology, Institute of Pharmacy, Ernst-Moritz-Arndt-University, Felix-Hausdorff-Str. 3, D-17489 Greifswald, Germany
[b]Institute of Biochemistry, Ernst-Moritz-Arndt-University, Felix-Hausdorff-Str. 4, D-17489 Greifswald, Germany
[c]Department of Chemical and Biological Engineering, State University of New York at Buffalo, 904 Furnas Hall, Buffalo, NY 14260-4200, USA

[$]Present address: Department of Biomolecular Chemistry, Leibniz Institute for Natural Product Research and Infection Biology, HKI, Beutenbergstr. 11a, D-07745 Jena, Germany

[*]Corresponding author: e-mail schweder@uni-greifswald.de,
telephone +49 3834 864212, fax + 49 3834 864238

This PDF file includes:

Introduction
Materials and Methods
Results
Table S1 to S3
Fig. S1 to S6
References

Introduction

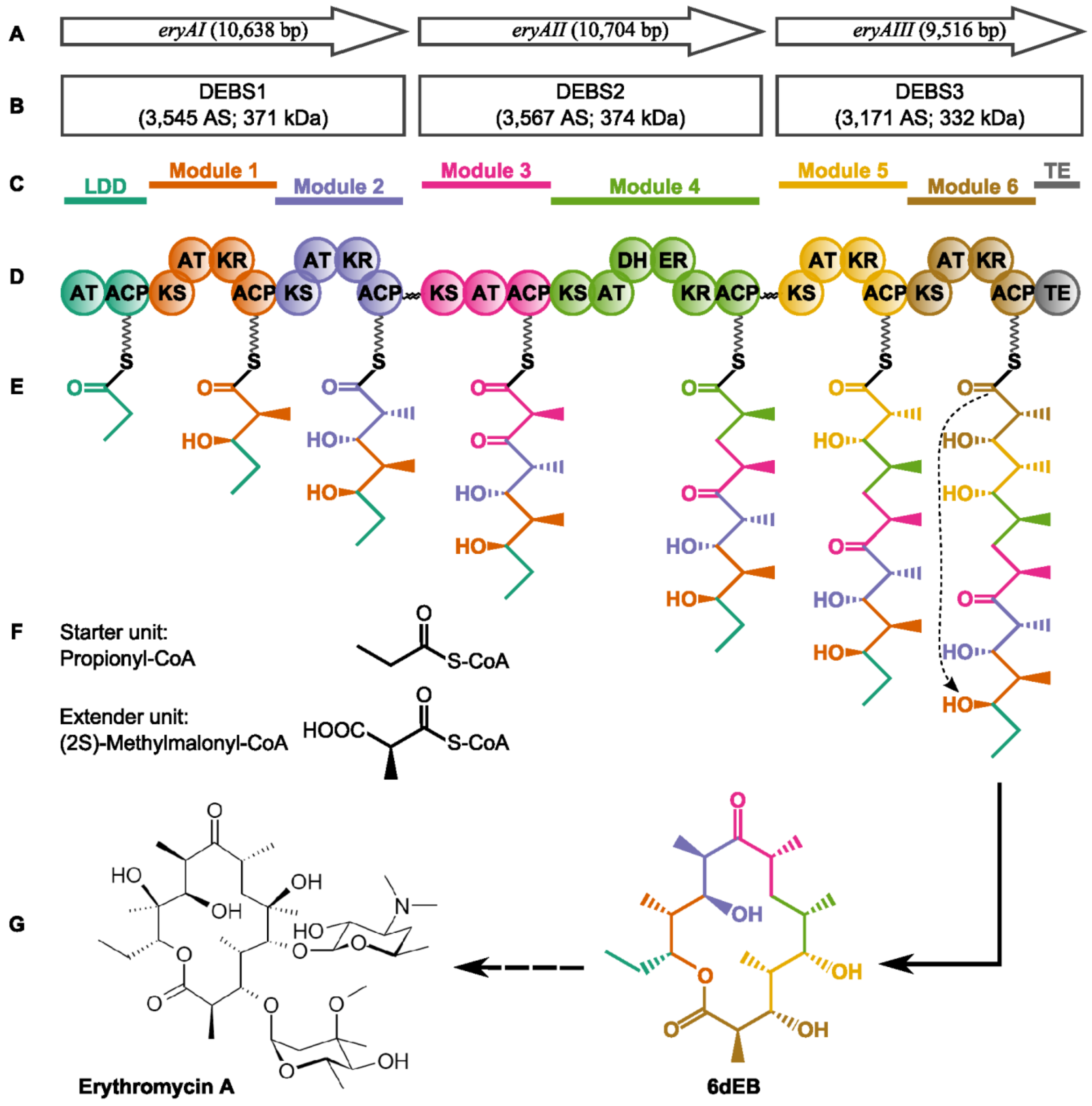

Fig. S1 Biosynthesis of 6dEB. The three *eryAI–III* genes (A) are coding for the DEBS1–3 proteins (B). These consist of one loading didomain (LDD), six modules for elongation and a termination (TE) domain (C) and are organized in catalytic domains (D). Biosynthesis (E) involves the assembling of the starter unit propionyl-CoA with six (2*S*)-methylmalonyl-CoA extender units (F). Depending on the presence of auxiliary domains, the keto-function remains unaltered or is partially or entirely reduced. The termination domain catalyzes the release of the metabolite from the enzyme by intra-molecular lactonization resulting in the macrolide 6dEB. Within the native host, *S. erythraea* 6dEB is subsequently modified to the antibiotic erythromycin (G).
Catalytic domains: AT, acyl transferase; ACP, acyl carrier protein; KS, ketosynthase; KR, ketoreductase; DH, dehydratase; ER, enoyl reductase; TE, thioesterase.

Materials and methods

Table S1 Oligonucleotide primers used in this study

Name	Sequence 5' → 3'	Usage
2355	CTAAAATTGGTTATGCACGACTCTAGCCTGCAGGTCGACTTAAACGCTAACGGTCAGC	Forward primer for Spec^{R}-cassette
5026	ACGCGGGGAGGCAGACAAGGTATAGGGCGGCGCCCAGGCTTCCATCTATCCGTC	Forward primer for *sfp*-front region
5016	CCACCTTCTCGCTGTCAGTCATATGTGTTTCCTCCTTCTATTTAGGGTTC	Reverse primer for *acoA*-promoter (*eryAII*)
5017	CTCTAAACGGGTCTTGAGGGGTTTTTTGTTTATTCAGTCAAACGATGCAG	Forward primer for *acoA*-promoter
5056	GAACCGGTCGCGGTCGTC	Reverse primer for *eryAI* probe
5072	CCGTTTAGAGGCCCCAAGGGGTTATGCTATCTAGATCGACTAGTTTAATCGCCGTCGAGCTCCCG	Reverse primer for 3‘-region of *eryAI*
5076	GAGAGCTTTGACAGGTCCGCCATATGTGTTTCCTCCTTCTATTTAGGGTTC	Reverse primer for *acoA*-promoter (*eryAI*)
5077	CGGCCGGGAGCTCGACGGCGATTAAACTAGTATGGAGGAAACACATATGACTGACAGCGAGAAGGTG	Forward primer for 5‘-region of *eryAII* (RBS)
5081	CTCTAAACGGGTCTTGAGGGGTTTTTTGTTTAAACACATATGACTGACAGCGAGAAGGTG	Forward primer for 5‘-region of *eryAII* (P_{acoA})
5082	GCTTGAAAACGCAATTCAGGCGGCCACTAGGTTTAAACACATATGGCGGACCTGTCAAAGCTC	Forward primer for 5‘-region of *eryAI*
5090	GTCATGCCGTTGTCACCGCTCATATGTGTTTCCTCCTTCTATTTAGGGTTC	Reverse primer for *acoA*-promoter (*eryAIII*)
5097	GAAAACGCAATTCAGGCGGCCACTAGGTTTATTCAGTCAAACGATGCAGAG	Reverse primer for *acoA*-promoter
5105	ACAGGAAGACGACGTTGC	Reverse primer for *eryAII* probe
5149	GGCCCCAAGGGGTTATGCTATCTAGATCGACTAGTTTACAGGTCCTCTCCCCCGCCG	Reverse primer for 3‘-region of *eryAII*
5150	CCGTAAGATGCTTTTCTGTGACTGGTGAGTGGGGCAGGGCGTGTACGCGG	Forward primer for 3‘-region of *eryAII*
5158	CATTCGGTGAATAAGGAAGCACCAAAAAGCTCGAGAAATTAAAGTAATAAAGCG	Reverse primer for Spec^{R}-cassette
5160	CATTCGGTGAATAAGGAAGCACCAAAAAGCTCGAGCGCAACGCAATTAATGTG	Forward primer for SSS-cassette
5161	CTAAACGGGTCTTGAGGGGTTTTTTGCTCGAGGAATCATCCAGCTGAATACG	Reverse primer for SSS-cassette
5206	GGCCCCAAGGGGTTATGCTATCTAGATCGACTAGTTGAACAGGCCTCGACGCGGCGCGACTGC	Reverse primer for 5‘-region of *eryAI*
5207	GACTCTCCCGAGCAGTCGCGCCGCGTCGAGGCCTGGACAGGGTGCTGGCC	Forward primer for 3‘-region of *eryAI*
5273	GCCTACTTCTCAACAGACGTCAGGTTCCAACTTTCACC	Forward primer for Cm^{R}-cassette
5274	CTCCACGGGGAGAGCCTGAGTTATTCAGGCGTAGCACCAG	Reverse primer for Cm^{R}-cassette
5276	CTTATTTCATTATGGTGAAAGTTGGAACCTGTGCACCTGGACGAGCTGTG	Forward primer for 3‘-region of *eryAI*
5277	GGATCCGTCGACTCTAGAGTCACGTTTACAGGTCCTCTCC	Reverse primer for 3‘-region of *eryAII*
5278	CGGGTCTTGAGGGGTTTTTTGTTTAAACCCCGGGCAGGCCGAGGCGGCAGG	Forward primer for 3‘-region of eryAII
5279	CACGTTCGGCCCTGCCGCCTCGGCCTGCCCGGGTCGATGCCGGCGTTCTCC	Reverse primer for 5‘-region of eryAII
5286	GCTTTTGATCACGGTTGAAATGATCGGATGAATTCTAGAAAAAAACCCCTC	Reverse primer for 3‘-region of eryAIII
5288	GGCCCCAAGGGGTTATGCTATCTAGATCGACTAGTTTAATCGCCGTCGAGCTCC	Reverse primer for 3‘-region of eryAI

Table S1 continued

Name	Sequence 5' → 3'	Usage
5308	GGCCCCAAGGGGTTATGCTATCTAGATCGACTAGTTTATGAATTCCCTCCGCCCAG	Reverse primer for 3'-region of *eryAIII*
5309	GCTTGAAAACGCAATTCAGGCGGCCACTAGTAGGCCTACGCGCTGGCGACCG	Forward primer for 3'-region of *eryAIII*
5310	GATCGAGCAGTTCGGTCGCCAGCGCGTAGGCCTCGACGCGGCGCGACTGC	Reverse primer for 5'-region of *eryAIII*
5411	GTATAGCATACATTATACGAACGGTAGGCCTGTCGAGGCCTCGCGACTGC	Reverse primer for Kan^R-cassette
5412	GAGGGGTTTTTTGTTTAAACGTGACTCTAGACCAAAGCATAAAAACTTG	Forward primer for Kan^R-cassette
5413	GTTATGCTAACTAGTATCGATATCGAATTCTCGAGCATATGTGTTTCCTCCTTCTATTTAGGGTTC	Reverse primer for *acoA*-promoter
5414	AATGTATGCTATACGAACGGTAGGCCTCGATTCAGTCAAACGATGCAGAG	Forward primer for *acoA*-promoter
5451	CAAAAAACCCCTCAAGACCCGTTTAGAGGCCCCAAGGGGTTATGCTATCTAGATCGACTAGATTATGAATTCCCTCCGCCCAGCCAGGCGTCG	Reverse primer for 3'-region of *eryAIII*
5452	AAACCCGGGAAACCGGGCTGCCGACGCTGCTGGTCAG	Forward primer for 3'-region of *eryAIII*
5453	CGTCGGCAGCCCGGTTTCCCGGGTCCATCGCCAGCGCCTCGCGCGGCGAG	Reverse primer for 5'-region of *eryAIII*
5454	CTAAACGGGTCTTGAGGGGTTTTTTGTTTAAACACATATGAGCGGTGACAACGGCATG	Forward primer for 5'-region of *eryAIII* (P_{acoA})
5455	GTTCGGCGGGGGAGAGGACCTGTAAACTAGTATGGAGGAAACACATATGAGCGGTGACAACGGCATG	Forward primer for 5'-region of *eryAIII* (RBS)
5464	CGACAGGAGCACGATCATGCGCACCCGTGGCCAAGGATCGCTTGTGCTCCG	Reverse primer for 3'-region of *pps* operon
5465	GCTATACGAACGGTAGGCCTCGAGGATCCGGATTAGCGGACAGAGGCC	Forward primer for 3'-region of *pps* operon
5466	GAACGGTAGGCCTCTAGAGTCAGATCGATACTAGTCGACAAAGCGTTATTACAG	Reverse primer for 5'-region of *pps* operon
5467	GCAATTATTACCTCCACGGGGAGAGCCTGACGTCGGACATGCCGAAGTGCTC	Forward primer for 5'-region of *pps* operon
5471	ATGCTAACTAGTTTAGCCCGCAAGCCCGGC	Reverse primer for *ery-orf5*
5472	GAAACACATATGAGCACCTGGCTGCGG	Forward primer for *ery-orf5*
5473	CCTGAGACGTCTACCCGCCCGGTC	Forward primer for 3'-region of *eryAIII*
5474	CACCCGTGGCCACCTACTATCAGTACC	Forward primer for 5'-region of *prp* operon
5475	CCTCGAGGATCCTGCAACAAAAAAAGCCGGAG	Reverse primer for 5'-region of *prp* operon
5476	AGGCCTACTAGTCTAGAGTACTGCTAGCTCATGATTTTGTTTGATATC	Forward primer for 3'-region of *prp* operon
5477	GAGCCTGACGTCAGCAAACGAAGCTGTGATG	Reverse primer for 3'-region of *prp* operon
5490	CTAATACGACTCACTATAGGGAGAGCGAGGCCTGCAACGAGGTC	Forward primer for *eryAI* probe with T7 promoter
5492	GCAGTGGCAGGGGATGGC	Reverse primer for *eryAIII* probe
5493	CTAATACGACTCACTATAGGGAGACGATCCGTTCGGCCGCCTGC	Forward primer for *eryAIII* probe with T7 promoter
5496	CTAATACGACTCACTATAGGGAGAGGAGAGCGACCGCATCAGGC	Forward primer for *eryAII* probe with T7 promoter

Cm^R: chloramphenicol resistance cassette; Kan^R: kanamycin resistance cassette; $Spec^R$: spectinomycin resistance cassette; SSS: $Spec^R$ flanked by two six-sites; Zeo^R: zeocin resistance cassette.

Table S2 Gradient profile applied for the analysis of 6dEB

Time [min]	Eluent A [vol%]	Eluent B [vol%]
0.00	95.0	5.0
2.50	70.0	30.0
9.00	40.0	60.0
10.00	5.0	95.0
12.00	5.0	95.0
12.10	95.0	5.0
17.00	95.0	5.0

Table S3 Source dependent MS parameters optimized for the analysis of 6dEB

Parameter	Value
Gas temperature (°C)	300.0
Gas flow (L/min)	10.0
Nebulizer (psi)	40.0
Sheath gas temperature (°C)	350
Sheath gas flow (L/min)	10
Capillary (V)	3000.0
Nozzle voltage	500.0

Construction of plasmids

All plasmids for integration of the *eryAI–III* genes are presented in Fig. S2. In order to chromosomally integrate the modified *eryAI* gene in the *srfA* gene locus, the Kan^R-cassette of the *srfA* deletion plasmid pJK93 was replaced by a $Spec^R$-cassette, amplified from pAMY-Spec with the primers 5158 and 2355. Both, pJK93 and the resulting PCR product were digested with *Xho*I and *Sal*I and ligated to give pJK94. This plasmid was then cut with *Spe*I and the 5'-region of *eryAI* was integrated via a "sequence and ligation independent cloning" (SLIC) method as described elsewhere (Li and Elledge 2007) after its amplification from cosmid P1394 with the oligonucleotides 5082 and 5206. Hereafter, the resulting plasmid was digested with *Pme*I and the *acoA* promoter, amplified from *B. subtilis* 168 with the primers 5076 and 5097, was integrated via the same method. In a next step, this plasmid was cut with *Spe*I and *Stu*I and the 3'-region of *eryAI,* obtained from P1394 by using the primers 5072 and 5207, was integrated. The resulting plasmid now contained a P_{acoA}-5'-*eryAI*-*Stu*I-3'-*eryAI*-T_{T7}-cassette. Restriction of this plasmid with *Stu*I led to a linear product with homologous regions to the *eryAI* gene allowing recombination with cosmid P1394 via the Red/ET method (gene bridges) according to the manufacturer's protocol. The resulting plasmid was named pJK119. In order to use the previously described optimized cloning protocol for rapid and multiple genome modification in *B. subtilis*, the *lox*-SSS-cassette (in which the $Spec^R$-cassette is flanked by two so called six-sites, which are in turn surrounded by two mutated *lox* sites) for marker removal and an enhanced transformation efficiency (Kumpfmüller et al. 2013) was used to replace the $Spec^R$-cassette. To this purpose, plasmid pJK119 was digested with *Eco*RI and *Swa*I and plasmid pJET-lox-SSS was cut with *Mfe*I and *Eco*RV. Ligation of the intended fragments resulted in plasmid pJK119c (Fig. S3).

For the integration of the modified *eryAII* gene in the *srfA* locus containing the P_{acoA}-*eryAI*-T_{T7}-operon, the $Spec^R$-cassette from pJK119 had to be replaced by a Kan^R-cassette. Since Red/ET cloning requires a selection marker switch and cosmid P1394 already contained a Kan^R-cassette, an additional Cm^R-cassette for selection in *E. coli* was amplified from pCUlac_pB with the primers 5273 and 5274 and integrated in pJK123, cut with *Aat*II, via SLIC to give pJK134. The following modifications of this plasmid were all performed via the SLIC method. First the 3'-region of *eryAI* was amplified using P1394 and the oligonucleotides 5276 and 5288 and integrated in pJK134, cut with *Aat*II and *Spe*I, thereby replacing the 5'-*srfA*-region (landing pad). Hereafter, this plasmid was digested with *Pme*I and fused with the 3'-region of the *eryAII* gene, obtained from P1394 after amplification with the primers 5277 and 5278. The resulting plasmid was cut either with *Pme*I/*Sma*I or with *Spe*I/*Sma*I. The *Pme*I/*Sma*I digested plasmid was ligated with the 5'-region of the *eryAII* gene, amplified from P1394 using the primer pair 5081/5279. The resulting plasmid was digested with *Pme*I for integration of the *acoA*-promoter, obtained from *B. subtilis* 168 with the primers 5016 and 5017. Hereafter, this plasmid was linearized with *Sma*I and fused with the *eryAII* gene using P1394 and the Red/ET method to give pJK139 harboring the final 3'-*eryAI*-T_{T7}-P_{acoA}-*eryAII*-cassette. In analogue, the plasmid restricted with *Spe*I/*Sma*I was again ligated with the 5'-region of the *eryAII* gene, but amplified from P1394 using the primer pair 5077/5279. This resulted in a 3'-*eryAI*-RBS-5'-*eryAII*-*Sma*I-3'-*eryAII*-cassette that could be used for Red/ET cloning with P1394 and gave pJK140 containing the final 3'-*eryAI*-RBS-*eryAII*-cassette. For chromosomal integration it was necessary to add the *lox*-SSS-cassette. Hence, pJK139 and pJK140 were digested with *Eco*RI and ligated with pJET-lox-SSS, cut with *Mfe*I, to give pJK139a and pJK140a, respectively.

The plasmid pJK191 was digested with *Sca*I and *Spe*I. Hereafter, this plasmid was cut with *Pme*I, dephosphorylated and ligated with the 3'- region of *eryAIII* after its amplification from P1394 with the primer pair 5451/5452, thereby adding the T7-terminator, and its phosphorylation. Analogous to the construction of pJ139 and pJK140, the resulting plasmid was digested with either *Pme*I/*Sma*I or *Spe*I/*Sma*I. Again, the *Pme*I/*Sma*I digested plasmid was ligated to the 5'-region of the *eryAIII* gene, amplified from P1394 using the primers 5453/5454. The resulting plasmid was digested with *Pme*I for integration of the *acoA*-promoter, obtained from *B. subtilis* 168 with the primers 5090 and 5017. Hereafter, this plasmid was also linearized with *Sma*I and fused with the *eryAIII* gene using P1394 and the Red/ET method to give pJK245 harboring the final 3'-*eryAII*-T_{T7}-P_{acoA}-*eryAIII*-T_{T7}-cassette.

The plasmid restricted with *Spe*I/*Sma*I was also ligated with the 5'-region of the *eryAII* gene, but amplified from P1394 using the primers 5453/5455. This resulted in a 3'-*eryAII*-RBS-5'-*eryAIII*-*Sma*I-3'-*eryAIII*-T_{T7}-cassette that could be used for Red/ET cloning with P1394 and gave pJK246 containing the final 3'-*eryAII*-RBS-*eryAIII*-T_{T7}-cassette.

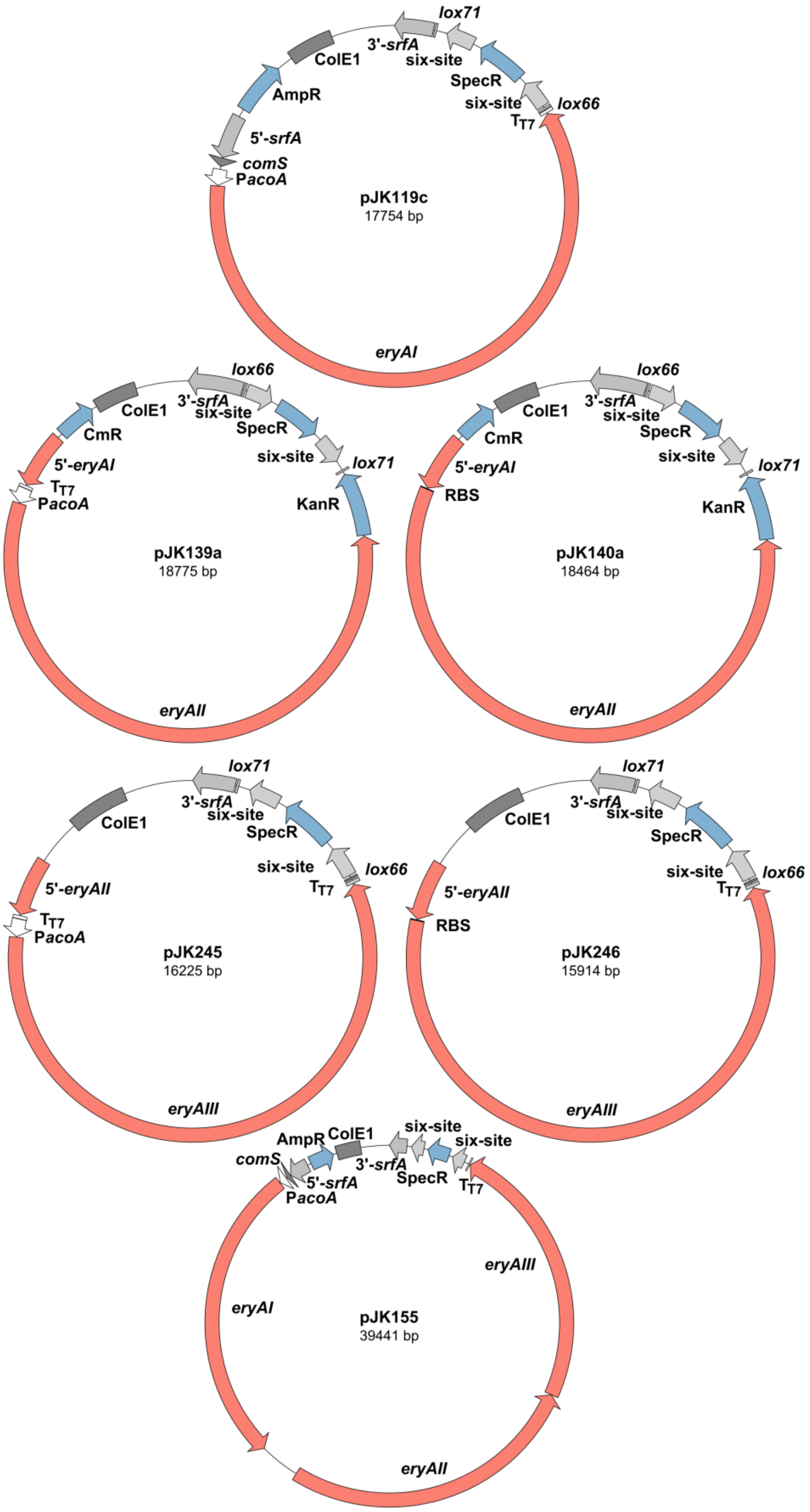

Fig. S2 Vector maps of the plasmids for the (stepwise) chromosomal integration of the *eryAI–III* genes.

The unmodified *eryAI–III* gene cluster was integrated in a single step. For marker removal a simple SSS-cassette (Spec^{R}-cassette flanked by two six-sites), obtained from pAMY-SSS with the primers 5160 and 5161 and cut with *Xho*I, was ligated into the *srfA* deletion plasmid pJK93, cut with *Xho*I and *Sal*I, thereby replacing the Kan^{R}-cassette to give pJK111. Hereafter, this plasmid was digested with *Spe*I and fused with the 3'-region of the *eryAIII* gene, amplified from P1394 with the primers 5308 and 5309, via SLIC. In a next step, the P_{acoA}-5'-*eryAI*-cassette was amplified using pJK119 as template and the primer pair 5097/5310 and integrated into the obtained plasmid, cut with *Stu*I. This plasmid was then used for Red/ET cloning with P1394 to give pJK155 which contained the final P_{acoA}-*eryAI–AIII*$_{nat}$-T_{T7} operon.
In order to simplify future expression studies, a plasmid for deletion of the *srfA* operon containing both a P_{acoA}-T_{T7}- and the *lox*-SSS- cassette was constructed. To this purpose, the *srfA* deletion plasmid pJK126, which already harbored the T7-terminator was digested with *Bam*HI and ligated with the *lox*-SSS-cassette obtained from pJET-lox-SSS, cut likewise, to give pJK218, thereby replacing the Nm^{R}-cassette. Hereafter, this plasmid was linearized with *Xho*I and fused with the *acoA*-promoter, amplified from *B. subtilis* using the primers 5413/5414, via SLIC to give pJK219. This plasmid was then used for the *ery-orf5* cloning. Therefore, the CDS was amplified from *Saccharopolyspora erythraea* DNA using the primer pair 5471/5472, digested with *Nde*I and *Spe*I and ligated with pJK219, cut in the same manner, to give pJK257. In order to chromosomally integrate the P_{acoA}-*ery-orf5*-T_{T7} operon behind the *eryAI–III* cluster located in the *srfA* gene
locus, pJK257 was digested with *Aat*II/*Nhe*I and ligated with the 3'-region of the *eryAIII* gene, amplified from pJK245 with the primers 5473/5286 and cut with *Aat*II/*Xba*I thereby replacing the 5'-*srfA*-region and resulting in plasmid pJK258.
For the deletion of the *ppsABCDE* operon, responsible for synthesis of the nonribosomal peptide plipastatin, plasmid construction started from pAMY-lox-SSS. To this purpose, the 3'-region of the operon was amplified from *B. subtilis* with the primers 5464/5465, digested with *Msc*I and *Bam*HI and ligated with pAMY-lox-SSS, which was cut likewise. Hereafter, this plasmid (digested with *Aat*II/*Spe*I) was ligated with the 5'-region of the *pps* operon, obtained with the oligonucleotides 5466/5467 and cut in the same manner to give pJK254.
The plasmid for deletion of the *prpBD* operon, responsible for propionyl-CoA utilization, was constructed likewise. pAMY-lox-SSS was cut with *Msc*I/*Bam*HI and ligated with the 5'-region of the *prp* operon, obtained from *B. subtilis* with the primer pair 5474/5475 and cut in the same manner. This plasmid was then digested with *Aat*II/*Xba*I and ligated with the 3'-region of the *prp* operon, amplified with the primers 5476/5477 and cut with *Aat*II/*Spe*I to finally give pJK260.
Furthermore, a plasmid for integration of a remaining Kan^{R}-cassette together with a removable, transformation-enhancing *lox*-SSS-cassette was constructed as follows. Plasmid pJK191 was digested with *Xba*I and ligated with the Kan^{R}-cassette, amplified from pMSE3 with primers 5411/5412 and cut likewise, to give pJK206.

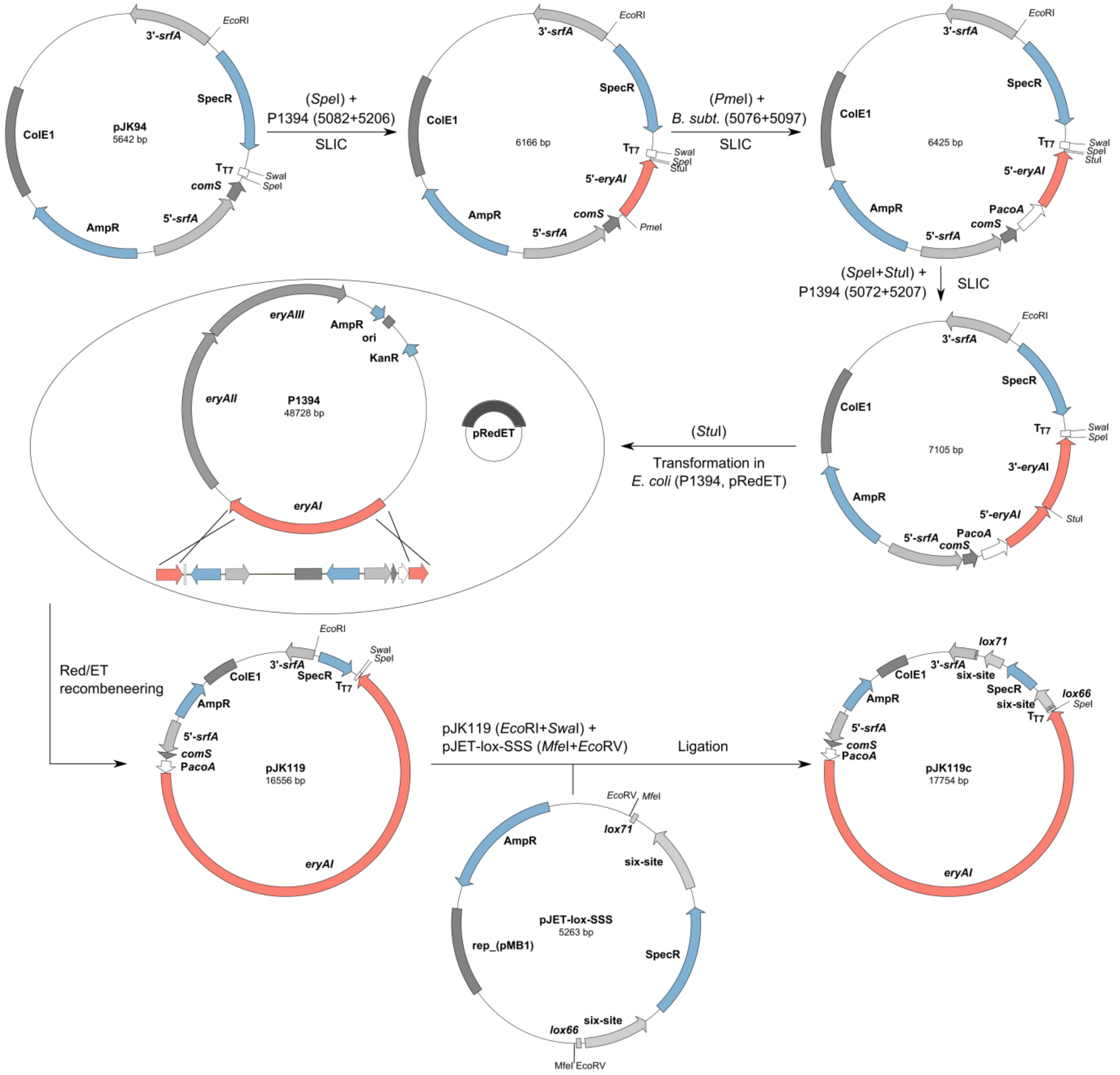

Fig. S3 Scheme of plasmid construction for the chromosomal integration of *eryAI*. Endonucleases for plasmid digestion, templates and oligonucleotides for PCR as well as the cloning method are specified.

Results

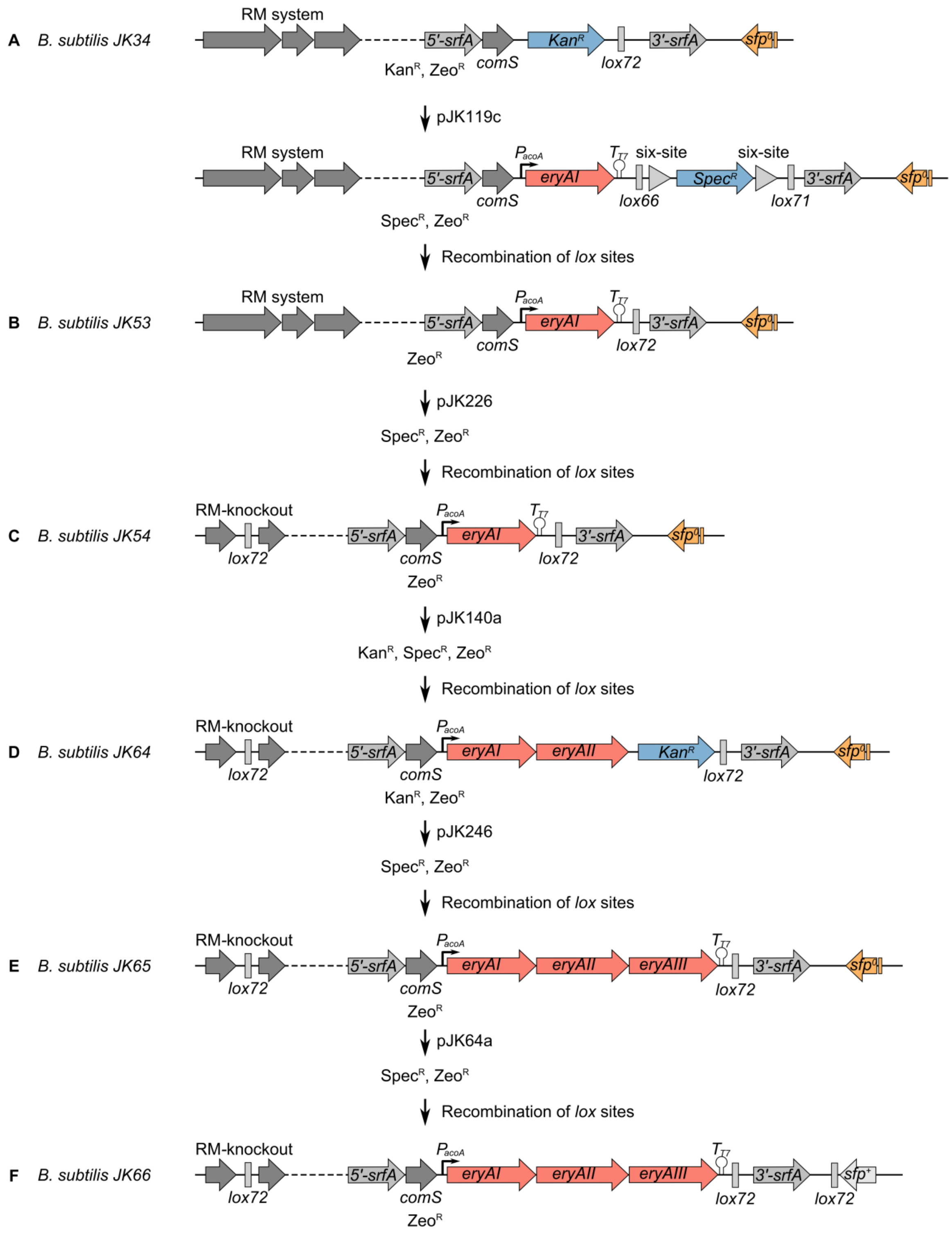

Fig. S4 Integration of the *eryAII–III* genes with optimized RBSs as one operon in *B. subtilis* JK54 under control of the *acoA* promoter. Schematic diagram (not to scale) showing the construction of *B. subtilis* JK66. **A**, the former *srfA* gene locus in the chromosome of *B. subtilis* JK34. Transformation of this strain with pJK119c and subsequent recombination of the *lox* sites via Cre resulted in *B. subtilis* JK53 (**B**). Deletion of the RM system via pJK226 resulted in *B. subtilis* JK54 (**C**). Transformation of this strain with pJK140a and subsequent recombination of the *lox* sites via Cre resulted in *B. subtilis* JK64, thereby replacing the T7-terminator of *eryAI* by an optimized RBS sequence (**D**). Chromosomal integration of the *eryAIII* gene via pJK246 resulted in *B. subtilis* JK66 (**E**). Finally, the frame shift mutated *sfp*^{0} gene was replaced by the active *sfp*$^+$ gene by using pJK64a to give *B. subtilis* JK66 (**F**).

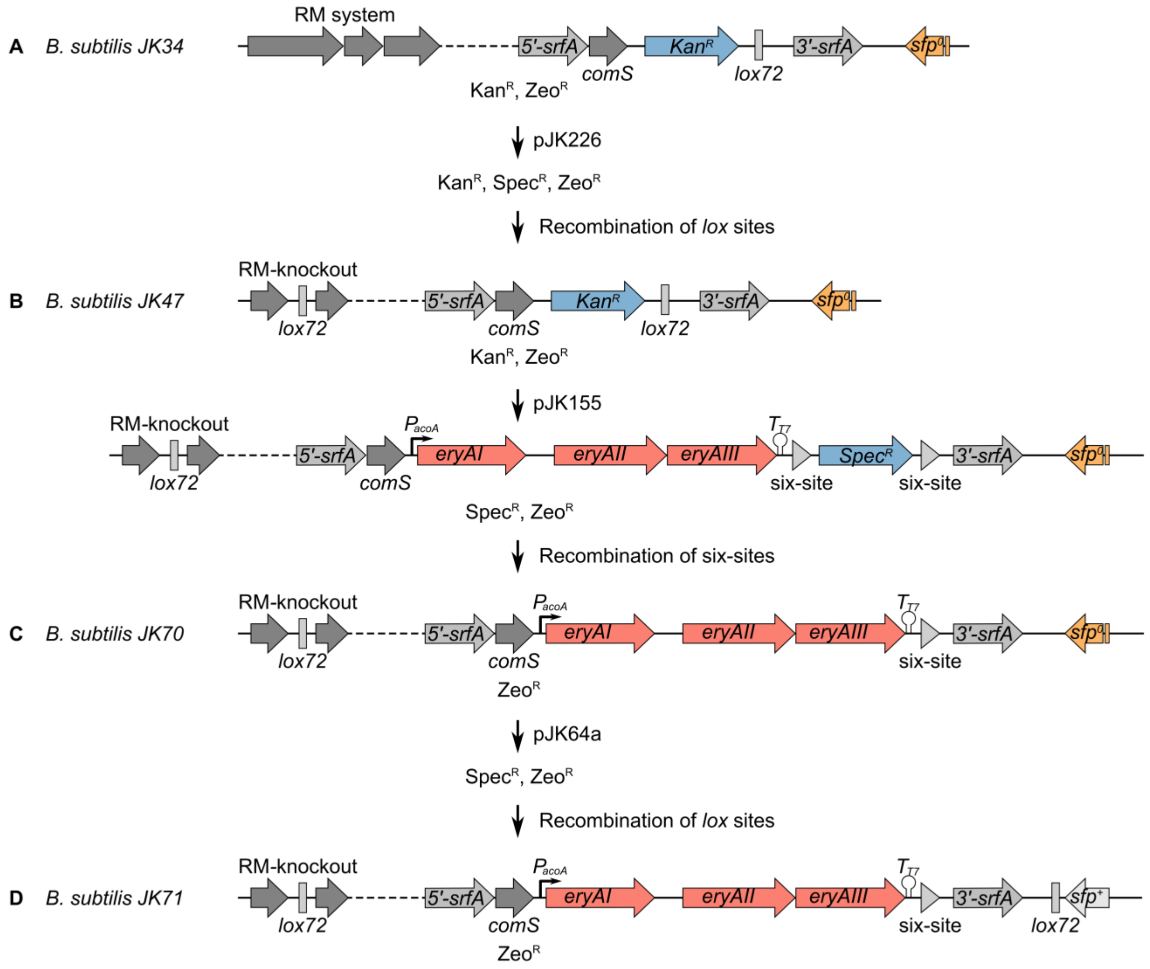

Fig. S5 Integration of wild-type *eryAI–III* operon in *B. subtilis* JK34 under control of the *acoA* promoter. Schematic diagram (not to scale) showing the construction of *B. subtilis* JK71. **A**, the former *srfA* gene locus in the chromosome of *B. subtilis* JK34. Deletion of the RM system by using pJK226 and subsequent recombination of the *lox* sites via Cre resulted in *B. subtilis* JK47 (**B**). Chromosomal integration of the wild-type *eryAI–III* genes via pJK155 resulted in *B. subtilis* JK70 (**C**). Finally, the frame shift mutated *sfp*^{0} gene was replaced by the active *sfp*$^+$ gene by using pJK64a to give *B. subtilis* JK71 (**D**).

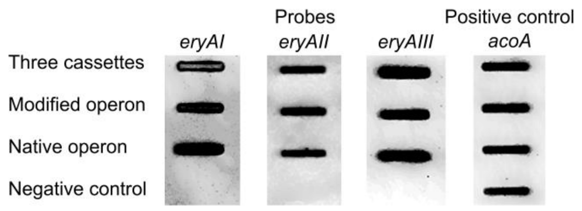

Fig. S6 Detection of *eryAI–III* transcription by slot-blot hybridization with specific probes and *acoA* as positive control in all three recombinant strains. The wt strain without the *ery* genes was used as negative control.

References

Kumpfmüller J, Kabisch J, Schweder T (2013) An optimized technique for rapid genome modifications of *Bacillus subtilis*. J Microbiol Methods 95:350–352. DOI: 10.1016/j.mimet.2013.10.003

Li MZ, Elledge SJ (2007) Harnessing homologous recombination *in vitro* to generate recombinant DNA via SLIC. Nat Methods 4:251–256. DOI: 10.1038/nmeth1010
